# Unusual CT Appearance of Bony Metastases from Malignant Peripheral Nerve Sheath Tumor

**DOI:** 10.5334/jbr-btr.1089

**Published:** 2016-11-24

**Authors:** Seema Doering, Cedric Boulet, Maryam Shahabpour, Johan De Mey, Michel Demaeseneer

**Affiliations:** 1 UZ Brussel, BE

**Keywords:** unusual, bony metastases, CT, malignant, bone tumors, malignant peripheral nerve sheath tumors

## Abstract

We present a case of bone metastases from a malignant peripheral nerve sheath tumor (MPNST). While multiple bone lesions typically are suggestive of metastatic disease, especially in combination with a primary tumor and positive PET, the appearance of lesions in this patient was quite atypical. We are not aware of any case of metastatic disease with such atypical *doughnut appearance* as in our case.

## Introduction

Bone metastases are common in many tumors. The diagnosis becomes highly suggestive, especially when they are multiple and PET positive. Differential diagnosis with multiple bone islands, tuberous sclerosis, sarcoidosis and myelofibrosis should always be kept in mind. Final proof can be obtained by biopsy. Metastases tend to have a focal or diffuse lytic or sclerotic aspect. In our case, the lesions appeared with a doughnut shape and we are not aware of any previous such atypical lesions.

## Case report

An 83-year-old man underwent surgical excision of a malignant peripheral nerve sheath tumor (MPNST) at the right knee at another hospital and was then referred to our hospital for further chemotherapy. Three months after the start of chemotherapy, the patient complained of severe pelvic and low back pain. A PET CT was performed which showed multiple bony lesions with a variable (moderate to high) degree of metabolic activity raising suspicion that these were metastases. The lesions, however, had a very unusual appearance and evolution as seen on CT.

**Figure 1:**
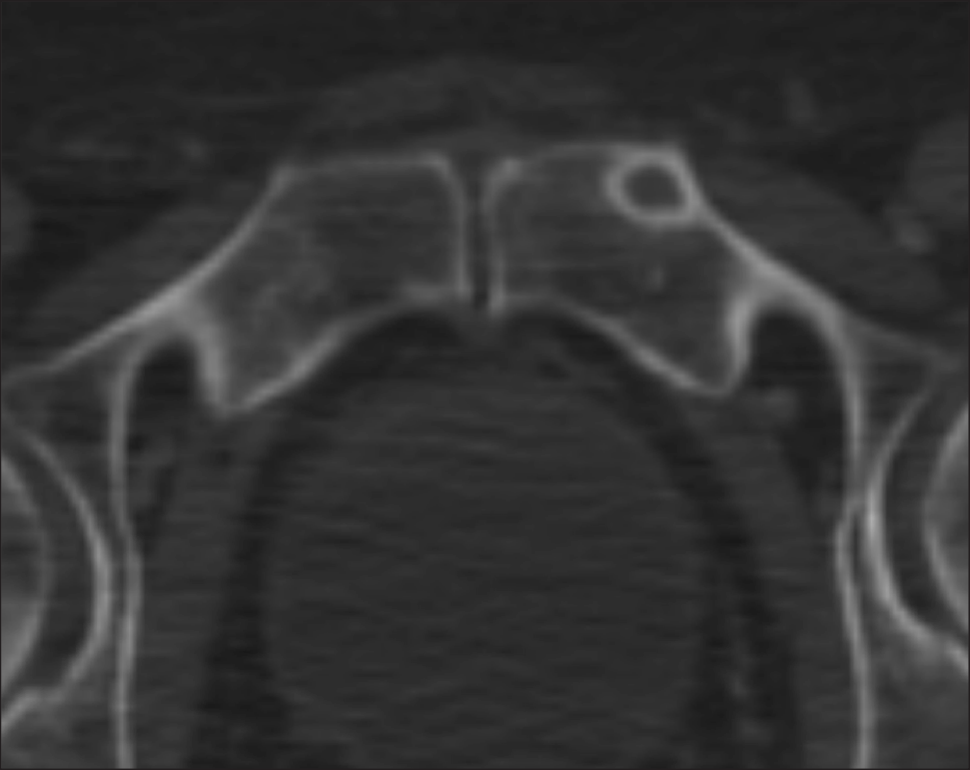
Bone metastasis arising from MPNST. The lesion has a central lytic area surrounded by a well-defined sclerotic lesion.

**Figure 2:**
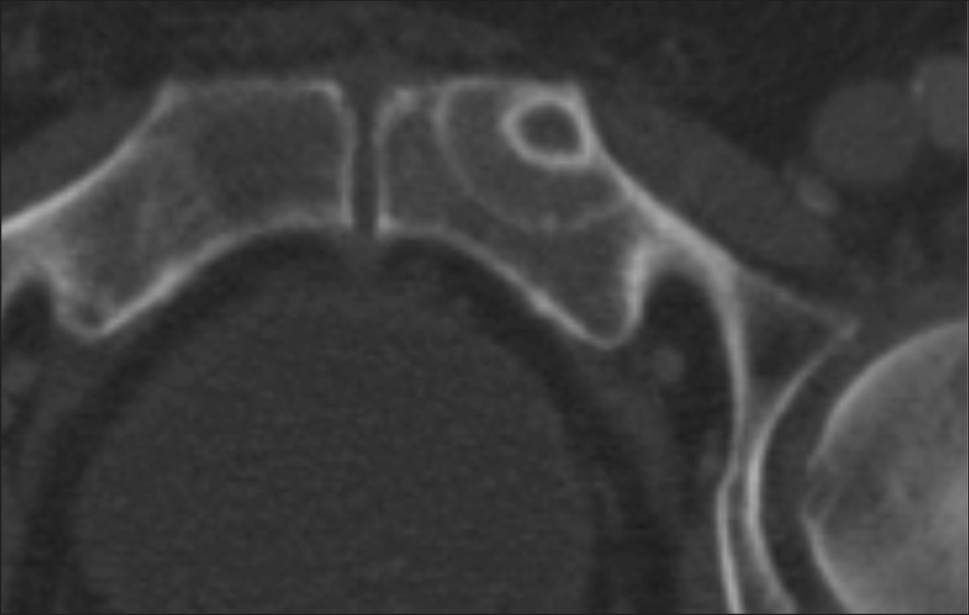
CT image of the same lesion as in Figure 1 after 7 weeks. Note the appearance of a halo with ground glass appearance around the initial lesion with a second thinner sclerotic margin giving it a ‘doughnut’ appearance.

Initially, the lesions presented as small rounded lytic lesions with a well-defined sclerotic margin (Figure 1). They exhibited a rapid growth but the initial appearance of central lysis and surrounding sclerotic margin was preserved. Follow up PET CT scan was performed after seven weeks to evaluate the response to chemotherapy. It revealed a second concentric band (halo) around some of the lesions. This band (halo) had a ground-glass appearance and was surrounded by a second sclerotic rim. This rim was thinner and less sharply demarcated than the more central rim (Figure 2). The multiple lesions were in different phases of evolution and as such had a different appearance at any given time.

**Figure 3:**
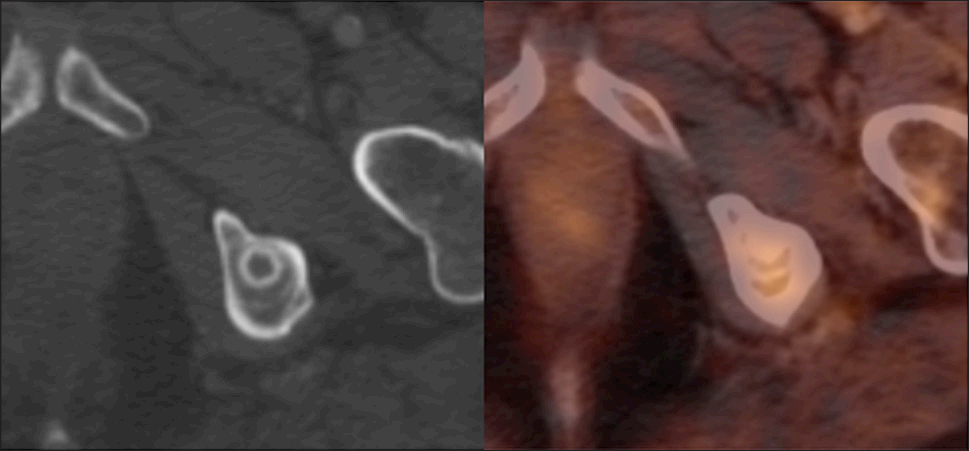
There is high uptake of FDG tracer on PET CT in this metastasis from a malignant peripheral nerve sheath tumor.

Presence of multiple lesions, a known primary tumor and moderate to high uptake of FDG tracer on PET scan (Figure 3) suggested metastatic disease, despite the unusual appearance of the lesions.

Other bony metastases were also evident on CT. Some were entirely sclerotic and a few had an ill-defined lytic appearance. Collapse of a vertebral body and fracture of the upper endplate of another vertebra due to metastases were also present.

**Figure 4:**
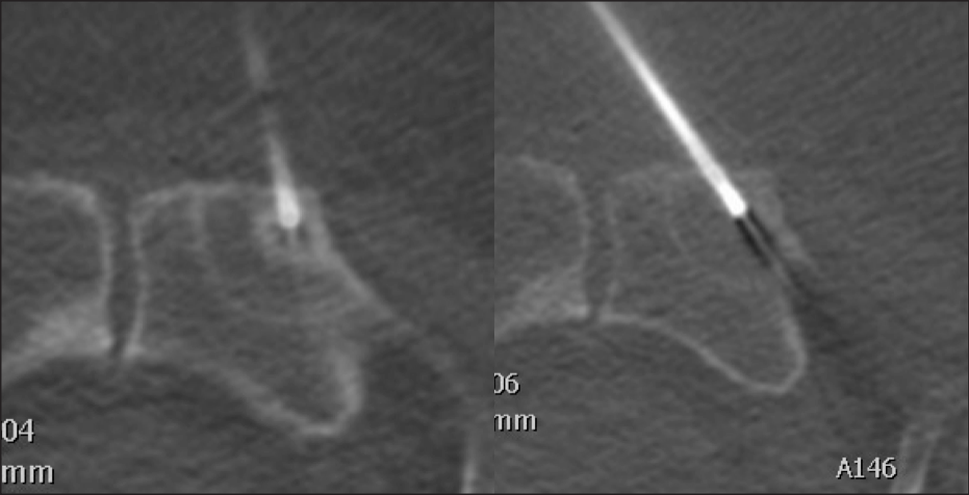
Biopsy performed from the central lytic area and from the surrounding halo from one of the metastases.

Bone biopsy of a lesion with the above described doughnut appearance located in the left pubic bone was performed. A biopsy was obtained in the central lytic area and another biopsy in the peripheral halo (Figure 4). Histological evaluation of both the samples showed infiltration of bone marrow by malignant spindle-shaped cells arranged neatly in bundles. Histological and immunohistochemical studies were compatible with metastases from a spindle cell tumor (in this case MPNST).

## Discussion

MPNST accounts for 5 to 10% of malignant soft tissue tumors and usually affects adults 20 to 50 years of age. A sarcoma is defined as a MPNST if it arises from a peripheral nerve, a preexisting benign nerve sheath tumor (neurofibroma) or demonstrates nerve tissue differentiation on histologic examination. Usually these tumors are aggressive and have a poor prognosis. Metastases most frequently involve lung, bone, pleura and retroperitoneum [1,2].

The most common malignant tumors that metastasize to bone are breast, lung, prostate, thyroid, and renal carcinoma. Bone reacts in different ways to different types of primary tumor cells. This may produce responses in the bone including lytic, sclerotic, or mixed response [3]. Solid cancers metastasize to bone by a multistep process that involves interactions between tumor cells and normal host cells. In the case of breast carcinoma, bone destruction is mediated by osteoclasts stimulated by local production of the tumor peptide parathyroid hormone-related peptide (PTH-rP), whereas prostate carcinomas stimulate osteoblasts to produce new bone [4].

Bony metastases may mimic almost any bone lesion [5]. Some slow-growing metastases may mimic a primary bone tumor with mineralization and sclerotic margins [6]. Metastases resembling certain benign conditions such as osteopoikilosis have been described [7].

Bony metastases in our patient do not resemble any known primary or secondary bone tumor, non-neoplastic bony lesion, or any other bone disorder. The presence of a sclerotic margin around a lesion is generally regarded as evidence of slow growth. In our patient, however, the metastasis showed rapid growth within a time frame of weeks.

Biopsy revealed the presence of malignant cells in both the center of the lesion as well as in the perilesional halo. It is quite unusual that progressive invasion of bone by malignant cells occurs in the form of a halo and with complete preservation of the initial sclerotic rim. We believe that the sclerotic margins around the central lytic area, as well as around the halo correspond to a bone reaction in an attempt to restrict the growth of the metastasis, and the rims do not actually contain malignant cells. A resection of the metastasis en bloc with histopathological analysis could shed more light on the actual composition of the different parts of the lesion. In our patient, resection of the metastases was not performed because it was clinically not indicated and would not affect prognosis of the patient.

Although several bone lesions can mimic bony metastases and vice versa, in our review of the literature we did not find any bony lesion with an appearance similar to the one we observed on CT in our patient. Whether this appearance can only be observed with metastases of MPNST is not entirely certain, but this may well be the case. A review of the clinical chart of the patient did not reveal any finding that could help explain the unusual appearance.

## Conclusion

In summary, we have demonstrated in our biopsy-proven case that a unique doughnut shaped metastatic pattern can exist in MPNST. Further study should investigate whether this can also be seen in other tumors or is typical for MPNST.
